# "Pray for Me"

**Published:** 1860-03

**Authors:** 


### "PRAY FOR ME."

There are two senses in which this very pretty little request is understood—the great majority of those making the request use it in the little sense. The exception in this, like most questions bearing upon our whole natures, is to understand and use it in the great sense.

In the first place, the very language is that of dependence or a sense of defficiency—need—in a word, something less than repletion or sufficiency. A state of attainment below our conceptions of completeness or competency. Something short of ability to answer our desire, to fully symbolize our ideal in the outward responsibilities and activities of life. Clearly teaching us that our hearts *must* first *conceive* before our hands *can* execute any work worthy the ambition of a human being.

In professional life there is a time and circumstance of being begotten, gestated, developed and born, all prior to the exercise of any thing that can be reckoned as matter of volition or duty upon the part of the individual. And not only so, but so far from our taking *any* responsibility in any of these minor deal conditions, they take place in absolute independence of our volition, for they are the points at which the forces upon which we are dependent culminate, and thus *produce* us professionally just as the creative and procreative forces *precede* and bring us *forth* naturally.

The how of all this would take the life-time of honest, earnest observation to elucidate in detail so as to sway the whole in any sense worthy of being termed *conviction*.

But we can not at first span the greatest extent of the most gigantic hand, let us not *refuse* to measure the pretty little span that can easily be effected with our chubby, infant thumb and finger, that if we will by quick succession of efforts, we may compass the whole distance that the great hand of the Brobdignag makes at *once*, in a much less space of time than would seem possible to the lazy objector who stands dreading to make any effort to advance for fear that he should fail.

In professional begetting, we have made patent to our consciousness the programme of natural begetting, &c., &c.

Now let us look it steadily in the front and see what it teaches.

1st. Our consciousness tells us that a new vibration has taken place among the elements of our perceptions, and arrests our attention to " divine " whither it tends. The exact direction, does not in an instant indicate clearly what ultimately will be settled upon, for the reason that the motions play about centers like circular waves in a pool, highly disturbed by a small body having been dropped into it. But keep up the closest watch and presently you will perceive that this first pebble was only "*avante courier*," to the corps that was to follow, and in due time indicate not only direction but every other specificity of the (to us) new individuality—circle or cycle of individuals, that the forces, part of which we ourselves are, were about bringing into the field of activities of the plane on which we stand.

At this point a fine opportunity is afforded to dilate fully upon the laws of living beings, but the flow of thoughts too abstruse for most minds to catch, must be checked and come more at the clearly defined; therefore let me say—our profession then has been begotten, gestated and born, as a distinct body. But its parts like those of the community of the race, are still in all these states or stages of *progress* or *regress*. Let us say that it has head, heart and lungs, hands—as necessary elements of its body proper. Instruments, tools and things may represent the means by which to handle principles and modes to useful ends.

That all this may be effected, this giant baby must be developed: which simply means that it must aggregate to itself the necessary constituents capable of nourishing it. No pabulum has as yet been found, chemically fitted, analytically to feed it up to a higher point than barely to enable it to use the surplus of elemental principles lodged in its tissues, by loss of its weight: thus living or rather languishing on its own substance, at the expense of size and weight; a condition only desirable as a compromise between immediate death and growth—in the hope that the disintegrating force shall have spent itself by the time a food properly combined of nutritious and effete substances so alternated as to afford the requisite stimulus, and at the same time present the analytical elements of growth to the organism has been secured. Laying us under the necessity of being always supplied with suitable at least, if not the most desirable preparations of these various elemental substances—food.

This prepares the way for me to say that fine thinking must first be fine feeding—as necessarily as fine workers must first be fine and analytical thinkers; demonstrating the close relation there exists between doing—working—and the high etherealism of the finest thought.

We are too prone to stand out against our highest interests by taking sides and debating every subject that is presented to us, at all touching upon the source of our thoughts; apparently determined to "account" for every thing that bubbles up in our hearts,—(showing us how imprisoned we are in a mere *secular* "commercialism" not having patience nor power to study out the time and only profitable "commerce" of heart and brain). Giving it a place in our old musty desks, to be sure the best we may have for the present for it, rather than simply to let some one who claims to have seen more and traveled farther than the masses, present us with something, give it a name and put it in an appropriate place; so that we could test by observation the fitness of the arrangement.

The first approach to the explanation of thought is clearest shown on the electrical plane of existences; to understand which in any satisfactory sense we must fulfill a few necessary conditions. These must be observed either by design or by fortuity, and noted in their proper time and place, to constitute them land marks by which to work out our problem.

If by accident—long intervals or short may intervene in the items, the very irregularity of which will complicate and render them less useful for our purpose; but better, much better have even these than none. If by design—let competent observers project their schemes never so wisely, each one will have ample opportunity to find fault, if not in the profession and exercise of that necessary element of all scientific research—*patience.*

Let us state a few facts bearing upon the point, under what circumstances the highest manifestations of the power of thought have displayed themselves. (*a*) In healthy persons, an empty or nearly empty stomach is a requisite of brilliant thought. (*b*) Etherially charged brain produces a very high mental stasis (instances of intoxication prove this). (*c*) Electrically charged brain eliminates high-toned and clearly defined thought. (*d*) *Fine* organizations eliminate the clearest thoughts in *elevated* places and *empty* stomachs, and will always have the power to catch and retain thought in a greater degree if they live regularly, are cleanly in personal habit, indulge much in a prayerful state of mind, never cut and but seldom trim hair or beard, and live much in the open air and seclude themselves from society a part of each day, for the exercise of mentally digesting and arranging what the mentals have gathered; exercise here as elsewhere is the law of *complete* development.

Very many who value themselves upon their genius and mental ability, have scouted the idea that a person can perform a work better under the conditions named than without their presence. But here as elsewhere the difference is more in the words used to convey the fact than the fact itself. For instance, some of the finest artists have the reputation of being any thing but men of prayer. Now let us see if they have not really prayed often and long, "with their all of desire," for certain attainments by the closest attention to what to them seemed the power to convey to them the capability to out-work their desires in their various departments? And rested not till they had produced by constant, earnest effort, in what was to their perception the best way, the work upon which their hearts had been set.

I do not say that a soul of genius can be satisfied with his productions, viewed in the light of his inmost conceptions of perfection. But I do mean to say his work does far surpass his who is the mere imitator of some one whom he looks up to as pattern; for the very reason that the desire of his soul is always leading him to surpass the outward and imperfect by working out the symbolism of the ideally perfect, that resides in the interior of all and any who have the slightest claim to "genius."

The genius represnts the Christian who prays for others as well as for himself; while the lame imitator personates him who has some one to do his praying for him, for which he is simple enough to give of his substance to sustain. As well might we entertain the proposition of some one to do our eating and breathing .for us, and save us the trouble for a few shillings, in the foolish hope of flourishing in vigorous growth; the utter impossibility of which would soon be made patent in our death struggle. For *action* is the law not only of *life*, but particularly and peculiarly of *growth*. The great incubus that has rested down upon us is consequent upon bad or wrong teaching; the setting up of unutterable things and thus discouraging the mind in place of suggestively leading it forth a step at a time, till it culminates in unheard of strength and manly vigor and becomes in its turn the leader of others into new fields of profitable effort, demonstrating that beautiful saying, "there is that scattereth and yet increaseth"—joyfully leaving the bunk imitator to prove that other part of the saying, "and there is that withholdeth more than is meet—*but* it tendeth to poverty."

Every *act is* and ought to be considered a prayer to the power we pay homage to:—It matters not how undeveloped or cultivated soever we may be. Those who deny this are like the primaries in all departments ever are, merely echoing automatically the sounds that they hear, till by habit they acquire a use of the organs of voice to such degere, as to let the specific tone belonging to the individual manifest itself so clearly as to prove it to be a distinct and special one, the product of their own organism. The discovery of which, usually results in use to excess of the newly discovered power, like the boy with a new whistle piercing the ears of all in reach of its odiously shrill note. If you wish to test it, all you have to do is to suggest that there is some other note or instrument more to your taste. Then you will perceive that you are at once set down in his estimation as one having "no music in your soul," for which he in magnanimity is ready to pity you.

I could give examples of the various sorts of prayer, but you will perceive that to fill the programme it would involve the full catalogue of man's aims and ends, and hence I will instance but a few as textual references.

All who utter selfish prayers are praying in the little sense. While those who are broad enough in their petitions to reach the whole race—profession, nation, state, county or town;—indicate a base broad enough to keep them from topping over for want of sustentation; or ever being so circumstanced as not to be able to find a work to do, to further the interest of some of the departments recognized in their platform; and thus utter their prayers in the great sense.

The specific reason for our being so careful how we let our aspirations escape us is, that we always in kind invite influences like the promptings within. For be it understood we are immersed in elemental influences that have a constant tendency to occupy and possess themselves of any unappropriated space in our mental receptacles. Therefore, it is of the highest importance for us to have all our powers exercised in the right direction early; thus pre-occupying the ground with the good seed, that will inevitably be taken possession of by the seeds of tares and noxious plants as matters of necessity, if neglected.

Let us then, gentlemen of the profession, as earnest seekers for the highest excellence in our activities, always strive to do our best every time we attempt to put forth our hands to express our skill; and we shall soon be as some have been astonished at the rapid advancement we shall make as individuals, and the most useful of professions.

A highly learned teacher in our profession has asserted that "every one has a right to buy his knowledge by his experience"—I would rather say he commits a *wrong* to community and his own soul if he refuses the testimony of *competent* witnesses—who stand thick on every hand to lead him from his blindness and errors to the beautiful platform of unmistakable usefulness. If we let our light shine without unnecessary obstruction, there will not long be difference of opinion among those worthy to take rank, as competent first class operators and teachers. "Freely ye have received"—let it be the ambition of us all as "freely to give," imitating the divine plan, even to the unkind and the unthankful, for the good of the whole; that our profession may soon attain such elevation, that none other than first rate good men can be called to exercise its noble functions, viz:

"Redeeming us from the curse of broken law."

Mathematical modes of demonstration are best understood in the light of such trains of thought as the following:

All of A can not express nor constitute B. But some of the elements of the two are used in common to symbolize the idea desired to be expressed—the only reason of which is that the real worlds of thought are as yet not open to the common perception by any but symbolic means. The real worlds—worlds of ideas, are of necessity spherical—spheres—and perfect in whole or in part as certain as the grains of seeds composing the full measure are perfect in specific form or developmental force. So that it does not matter at what point on a sphere the advent is made, it is capable of being a sure starting point from which to survey and calculate relations. This must be so or the pupil could never transcend the master, into whose trammels he might have fallen. But that pupil can transcend masters in the aggregative great sense, nothing can possibly be more absurd. For as in procreation many elements that do not inform us from whence they come, go to make up the whole of our individuality ; so many entities and mental existences inflow to our personal perceptions that may never have existed or been awaked to action, in the consciousness of those we call our teachers or masters, but were aggregated from the spheres for the first in our own cases, without notifying us whether they were now really for the first time nucleated and tied down to symbolic harness to be driven by any one into whose hands the rein might by fortuity fall. So that each seat of affection is thrilled with pleasure when *it* first is cognizant of new ideas or combinations of ideas ; thus constituting the highest inducement to cultivate such states of induction for the purpose of letting the light shine. It matters not to the soul made happy by the blessed presence of living thought and perception, whether that particular thought or vibration ever stirred or ever will stir any other soul, mind or spirit in the universe, so long as it has fulfilled its mission in his particular case, any more than the satiated and replete stomach cares to inquire how many appetites the elements composing the repast just finished, had ministered to in the great round of aggregation and segregation of elemental changing substances. The *waste* of one form of living manifestation being but the constituent part of *food* to some other in the grand scale of the round of being,—exhibiting economy in the great sense, where narrowness of interpretation, could see nothing but the most improvident lavishment on one part and the most unjust and absolute prohibition and exaction on the other—to make up the balance.

Thus mathematical modes of teaching show us clearly that if there were no sources for advancement for us but those imprisoned in the means and minds of professional teachers, that we should soon be at a loss for even the ordinary degree of intelligence. For it can not be denied that all such teachers are liable to forget, and are not at all times in possession, in the communicative sense, of all that they may have been taught or known. So that thus limited we must retrograde in the main, instead of advancing; to avoid which we must keep our minds open toward the educating and growing, so that we may be less impoverished with our forgettings. And let the legitimate exchange of the various riches of our mental stores, advance from point to principle, forgetting *detail* but gaining the real elements of all specialities in the attainment of those that belong in the great sense to the whole of our world of scientific labor.

The great lesson to be drawn from our experience in life is, that we should not let the mode, manner or law of procedure, that properly belongs to some of the branches of scientific research, so overslaugh and possess the mind as to destroy the ability to look at the universe in any but a one-sided direction, and with the oblique light necessary to the clear definition of some of the less important, though beautiful, markings of her exterior.

Specialities and specialists are of course very desirable in their place, and to such an extent has this possessed the minds of men in the various departments of scientific research that, the pure mathematician on the discovery of a new object, puts his mathematical calipers upon it, and at once, as he thinks, determines its exact mark; mathematically exact value and sets it down as irrefragable testimony as to the value of that particular object, regardless of the class, order, general species or variety to which it belonged. Let this suffice as to the method of dealing with specialities by specialists; and come at once to the statement, that all things in time, place and action, will be fitting or unfitting, complete or deficient, just in the ratio of the ability of the observer to comprehend their *relations*. Therefore, if we would make real advancement, we must treat of things in the *various* lights of science, before we are entitled to mark, number and put them away for use. Not caring to follow the beaten track of faculties of institutions of learning, and publishers of serial works or rather *books*, by saying to the pupil, this belongs to Prof. so and so's chair, or this will be elucidated and dwelt demonstratably upon in such and such a work we are about to or have just issued for the benefit of all inquirers, at the very reasonable price of —— dollars and —— cents. But in the exercise of our free franchise, take up and dwell upon any and all subjects that may present themselves; even at the risk of being dubbed desultory—fanatical—enthusiastic—or any other odious name that the dullard imprisoned in his teachings may see fit to apply to us; and reap the rich harvest of freshness that springs up in a fertile soil, not trodden to mud, dust or filth, by the ease-loving, swinish old fogy that stands at the crossing to deter all from any but *his* particular way to be traveled, assuming that all that is worth knowing or professing is in the line of observation pioneered by our venerable antecedents, of whom he is the only legitimate representative. And lest we deny it produces an old piece of mummy cloth or other equally attractive piece of stuff marked with Hieroglyphical characters he can not decipher, and in triumph says: "there is my diploma."

This last remark will be of use to us if we look at it a moment, for it exhibits to us the object of obtaining a diploma that really is too often met. Mere show, instead of patiently plodding or moving with celerity along the pathway of investigation, for what knowledge is to be gained of a useful nature, rather than the mere credit of having paid so much money and spent so much time in such and such an institution, in consideration of which none but *men* can refuse to sign, seal and deliver the parchment, setting forth the qualities supposed to constitute worthiness for such a mark of fellowship. Nay, gentlemen, the real diploma resides in heads, hearts and hands, of all who are in any sense qualified for any true and pleasant fellowship on this or any plane of social duty. General truths, covering all special truth or truths of detail, to fully set forth which would require more time, ability and patience than can be commanded on this occasion. O.

